# Whole genome characterisation of quail deltacoronavirus detected in Poland

**DOI:** 10.1007/s11262-019-01639-1

**Published:** 2019-02-13

**Authors:** Katarzyna Domańska-Blicharz, Maciej Kuczkowski, Joanna Sajewicz-Krukowska

**Affiliations:** 1 Department of Poultry Diseases, National Veterinary Research Institute, Puławy, Poland; 2 Department of Epizootiology and Clinic of Bird and Exotic Animals, Faculty of Veterinary Medicine, Wroclaw University of Environmental and Life Sciences, Wroclaw, Poland

**Keywords:** Deltacoronavirus, Quail, Genetic characterisation, Poland

## Abstract

Quail deltacoronavirus (QdCoV) described for the first time in the United Arab Emirates in 2018 belongs to the same deltacoronavirus species as viruses discovered in swine and tree sparrows. The full-length genome of QdCoV detected in quails with enteritis in Poland has similar organization as Middle Eastern viruses although there is no NSP7c gene. The overall degree of nucleotide sequence identity was 92.4–92.6% between Polish PL/G032/2015 and Middle Eastern UAE-HKU30 QdCoV isolates. The sequences of the individual genes show similar nucleotide identities in the range of 91.4–94.7% with the exception of the S gene with lower identity of 85.6–85.7%. The most variable part of the S gene is its fragment encoding the N-terminal domain of the S protein which is responsible for receptor binding. The amino acid homology in this region between PL/G032/2015 and UAE-HKU30 QdCoVs was 74.5–74.7%. In contrast, the C-terminal domain of the S protein which is responsible for membrane fusion had an amino acid homology of 96.9%. In the phylogenetic tree, PL/G032/2015 branched separately but clustered with the UAE-HKU30 QdCoV isolates. These data suggest that PL/G032/2015 could be a new genetic/serologic variant of QdCoV.

**Electronic supplementary material:**

The online version of this article (10.1007/s11262-019-01639-1) contains supplementary material, which is available to authorized users.

Coronaviruses (CoVs), enveloped, positive-sense ssRNA viruses, belong to the subfamily *Orthocoronavirinae*, family *Coronaviridae*, suborder *Cornidovirineae*, in the order *Nidovirales. Orthocoronavirinae* are further divided into four genera *Alpha-, Beta-, Gamma-*, and *Deltacoronavirus* [9]. Generally, alpha- and betacoronaviruses infect humans and domestic animals while gamma- (gCoVs) and deltacoronaviruses (dCoVs) are associated mainly with avian but also mammalian hosts [12, 19]. The avian representatives of the *Gammacoronavirus* genus recently assigned to the separate *Igacovirus* subgenus are infectious bronchitis virus (IBV) and genetically similar viruses isolated from other domesticated galliformes [2, 8, 11]. The *Deltacoronavirus* genus, subdivided into four subgenera, currently comprises seven species, including six bird coronaviruses and one pig coronavirus, but deltacoronaviruses have also been identified in Asian carnivores [6]. The Old World quails (*Coturnix coturnix japonica*) seem to be susceptible to infection with two genera of avian coronaviruses, gCoV as well as dCoV [4, 16–18]. Recently, quail deltacoronavirus (QdCoV) has been reported in fecal samples of five quails sampled in the United Arab Emirates [10]. Phylogenetic analysis of their full genomes revealed that QdCoV UAE-HKU30 belongs to the same CoV species as porcine deltacoronavirus (PdCoV) HKU15 and sparrow deltacoronavirus (SpdCoV) HKU17 within *Buldecovirus* subgenus, suggesting transmission between avian and swine hosts. Moreover, most probably this quail dCoV originated from recombination between PdCoV/SpdCoV and munia deltacoronavirus (MundCoV) HKU13 [10]. This study presents the genetic characterization of deltacoronavirus detected in diseased quails in Poland.

In February 2015, an acute enteritis was reported on a quail farm located in the southeastern part of Poland, in Dolnośląskie province. The farm consisted of one house in which quail-layers and their progenies were housed. The building housed over 15000 layers reared in small sectors/boxes for 350 birds. Disease symptoms were reported in quails from one sector only. Firstly, short-lived diarrhea appeared in the parents, but subsequently severe symptoms were observed in offspring. Hatched chicks were weak; watery diarrhea started on days 2–4 of life, lasted about 2 weeks, and was accompanied by an elevated mortality of up to 3–10 birds per day. The birds were treated with antibiotics but without any beneficial effects and died at day 10–11 of life. Later on, increased embryo mortality was also noted. After the disinfection of the house, the problem did not reoccur.

Nucleic acids were extracted from intestines of dead 10- to 11-day-old quails and tested for the presence of enteric viral pathogens of poultry including parvovirus, astrovirus, adenovirus, and rotavirus [5, 7, 14, 20]. The samples were also checked for coronavirus presence using primers specific for the RdRp gene fragment of all CoVs and for untranslated regions (UTRs) at 5′ and 3′ genome ends of gCoVs [1, 2, 13]. The RT-PCR results revealed that tested samples were positive for coronavirus but negative for gammacoronavirus. Obtained bands were sequenced and blast (blastn) analyzed. The nucleotide sequence of partial RdRp gene of coronavirus detected in diseased birds revealed 93.5% identity with the quail deltacoronavirus UAE-HKU30 1101F strain (LC364346). The detected virus was named QdCoV/PL/G032/2015 and its complete genome was generated using Illumina MiSeq technology (Illumina Inc, San Diego, USA) offered by Department of Microbiology, National Veterinary Institute (SVA), Uppsala, Sweden. The sequencing reads were trimmed and de novo assembled into contigs, with which blast analysis against a virus database was performed. The reads were also mapped to available reference sequences and then consensus sequence was extracted. The obtained complete genome sequence of the virus was deposited in the GenBank database under the accession number MH532440. The full-length genome consists of 25881 nt and has the following structure 5′UTR-ORF1a/1b-S-E-M-NS6-N-NS7a-NS7b-3′UTR. The lengths of respective regions/genes and their putative products are shown in Table 1. Similarly to UAE-HKU30 QdCoVs, one ORF (NS7a) overlaps with N but in contrast to them only one additional ORF (NS7b) is present downstream of N, there is a lack of ORF coding nonstructural NSP7c protein.

**Table 1:** Regions/genes positions and lengths (nt and aa) of the QdCoV/PL/G032/2015 strain

Region/gene	5′UTR	ORF1ab	S	E	M	NS6	N	NS7a	NS7b	3′UTR
Position	1–495	496–19277	19259–22789	22783–23034	23027–23680	23680–23961	23986–25011	24080–24682	24995–25429	25430–25881
Length (nt)	495	18782	3531	252	654	282	1026	603	435	452
Length (aa)	–	6260	1176	83	217	93	341	200	144	–

The phylogeny based on the complete genome sequence revealed that the QdCoV/PL/G032/2015 strain formed a common branch with other quail UEA-HKU30 dCoVs recently identified in the United Arab Emirates, close to PdCoV HKU15 and SpdCoV HKU17 (Supplemental Fig. 1). Sequence analysis showed that the QdCoV/PL/G032/2015 strain shared nucleotide identities of 92.4–92.6% at the complete genome level with QdCoVs from the Middle East (Table 2). Nucleotide and aa sequence identity values of ORF1a/b gene between the QdCoV/PL/G032/2015 and UEA-HKU30 dCoVs were in the range 93.0–93.5% and 96.2–96.5%, respectively. Over 230 substitutions and one deletion of three aa (^759^KPD^761^) at positions 759 and 761 were observed between them (numbering refers to the respective protein of the Polish QdCoV). Based on the S gene, the QdCoV/PL/G032/2015 strain is also most closely related with Middle Eastern quail dCoVs but with rather low homology of 85.6–85.7% and 85.4–85.5% at nucleotide and amino acid sequence, respectively. In the phylogenetic tree constructed using nt sequences of the S gene, the QdCoV/PL/G032/2015 strain grouped with other QdCoV, close to MundCoV HKU13 (Fig. 1). 147 amino acid substitutions were observed in the S gene sequence of Polish quail dCoV strain when compared to isolates from Dubai area. Additionally, the QdCoV/PL/G032/2015 strain contained numerous insertions, i.e., four amino acids (^96^KQPE^99^) between position 96 and 99, 11 amino acids (^360^GNNISFYTTPA^370^) between position 360 and 370, two amino acids (^112^TN^113^) between position 112 and 113, and three insertions of single amino acid: N at the position 233, S at the position 414, and R at the position 1171. There were also two deletions of single amino acid, one (N) at the position 291 and the next (R) at the position 353. All mentioned insertions and deletions made the S gene longer by 18 amino acids in comparison to quail dCoV UAE-HKU30 strains. Interestingly, most of the amino acid changes were located in the N-terminal domain of S protein responsible for receptor binding. In turn, C-terminal domain of S protein responsible for membrane fusion seems to be more conserved. Deduction of the cleavage site is rather problematic as the furin could recognize different amino acid patterns; however, the putative cleavage site was deduced between position 691 and 692 just after the probable furin recognition pattern (^687^RKGGR^691^) according to the rules described recently [15]. The homology of the region preceding the putative cleavage site (amino acids 1-691) was 74.5–74.7% in contrast to the region behind this site (residues 692–1178) homology of 96.9%. The structural E gene of Polish QdCoV revealed identity with QdCoV UAE-HKU30 strains at the level of 93.3–93.7% and 98.8% at nucleotide and amino acid sequences, respectively. Only one amino acid (N for D) at the position five was changed in comparison with Middle Eastern QdCoVs. In the M gene, the Polish QdCoV strain had 91.4–91.6% and 93.5% nt and aa sequence identity, respectively. 14 amino acid substitutions were observed in QdCoV/PL/G032/2015 strain. In turn, the structural N gene seemed to be less different between compared QdCoV strains as nucleotide and amino acid sequence of the Polish strain had 94.4–94.7% and 97.9–98.8% identity, respectively, to QdCoV identified in the United Arab Emirates.

**Fig. 1:**
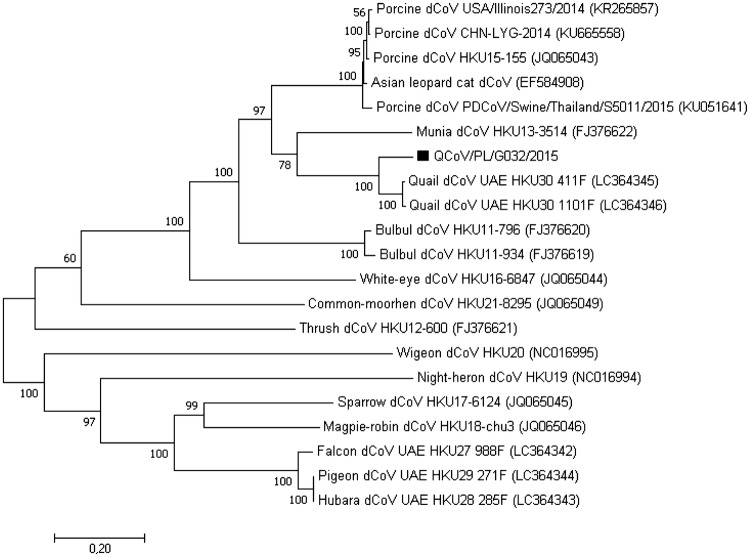
Phylogenetic analysis of the S gene of QdCoV/PL/G032/2015 strain from this study and other deltacoronaviruses. The tree was constructed using MEGA 7 using the neighbor-joining method and 1000 bootstrap replicates (bootstrap values shown on the tree). The scale bar indicates the number of nucleotide substitutions per site. GenBank accession numbers of the sequences are indicated in the parentheses. QdCoV/PL/G032/2015 strain determined in this study is marked with a black square

**Table 2:** Sequence identity of the complete genome and individual genes and proteins of dCoV/Q/PL/G032/2015 to other deltacoronavirus strains (in bold-the highest nt and aa identity)

	Genome	RdRp		S		E		M		N	
		nt	aa	nt	aa	nt	aa	nt	aa	nt	aa
Porcine_dCoV_HKU15_strain_HKU15-155_(JQ065043)	81.7	83.9	89.9	68.1	71.6	88.9	94	88.1	88.9	88.3	89.8
Porcine_dCoV_PDCoV/USA/Illinois121/2014_(KJ481931)	81.7	83.9	89.9	68.2	71.5	88.5	94	87.8	89.4	87.7	89.2
Porcine_dCoV_CHN-LYG-2014_(KU665558)	81.7	83.7	89.7	68	71.6	89.3	94	88.4	89.4	88	89.8
Asian leopard cat dCoV Guangxi/F230/2006 (EF584908)	n/a	n/a	n/a	70.1	66.4	88.8	94	88.1	89.4	87.7	89.2
Sparrow_dCoV_HKU17-6124_(JQ065045)	79.5	84.4	90.7	48.7	41.9	77.8	82.9	83.8	87.1	88.5	90.6
Magpie-robin_dCoV_HKU18-chu3_(JQ065046)	68.1	73.2	79.2	48.9	42.4	75.8	73.2	67.3	70.2	72.7	76.9
Munia_dCoV_HKU13-3514_(FJ376622)	70.1	72.4	78.2	66.6	70.2	69.8	68.3	65.4	72.9	69.1	75.4
Bulbul_dCoV_HKU11-796_(FJ376620)	67.5	70.4	75	63.4	65.9	69.8	74.4	68.6	71.1	70.2	71.9
Bulbul_dCoV_HKU11-934_(FJ376619)	67.5	70.3	74.9	63.5	65.5	69.8	74.4	68.3	71.6	69.9	72.2
Thrush_dCoV_HKU12-600_(FJ376621)	66.4	70	74.6	50.8	45.7	72.2	76.8	70.9	73.9	75.8	78.7
White-eye_dCoV_HKU16-6847_(JQ065044)	66.6	69	73	59.7	59.8	71.8	74.4	71.8	74.8	74.1	74.8
Common-moorhen_dCoV_HKU21-8295_(JQ065049)	61.7	65.4	68.5	53.5	49.4	63.2	63.4	52.4	14.3	61.5	59.8
Wigeon_dCoV_HKU20_(NC_016995)	52.5	53.1	50	48.5	40.9	47	36.3	57.1	53.2	52.4	50.1
Night-heron_dCoV_HKU19_(NC_016994)	53.4	54.1	51.3	46.7	40.4	49.2	36.1	58.4	53.2	50.5	50.9
Quail_dCoV_UAE-HKU30 411F (LC364345)	**92.6**	**93.5**	**96.2**	**85.7**	**85.4**	**93.3**	**98.8**	**91.6**	**93.5**	**94.4**	**97.9**
Quail_dCoV_UAE-HKU30 1101F (LC364346)	**92.4**	**93**	**96.5**	**85.6**	**85.5**	**93.7**	**98.8**	**91.4**	**93.5**	**94.7**	**98.8**
Pigeon_dCoV_UAE-HKU29 271F (LC364344)	65	69	74.1	49.4	42.2	75.8	78	70.5	72.9	72	73.5
Hubara_dCoV_UAE-HKU28 285F (LC364343)	65	69	74.1	49.3	42.2	75.8	78	70.3	72.9	72	73.5
Falcon_dCoV_UAE-HKU27 988F (LC364342)	65	69	74.1	49.6	42.3	76.2	78	70.9	73.4	71.4	73.2

Determining the similarity of nonstructural genes encoding in the 3′ portion of the genome is not so straightforward, primarily because the Polish strain does not have NS7c present in the Middle Eastern QdCoV UAE-HKU30 strains. Based on the NS6 gene, compared to QdCoVs UAE-HKU30 isolates, the QdCoV/PL/G032/2015 had 91.8% and 87.4–88.6% nucleotide and amino acid sequence identity, respectively. 10 and 11 aa were changed in the Polish QdCoV strain in comparison to the Middle Eastern isolates. In the NS7a gene, the QdCoV/PL/G032/2015 shared 95.1–95.5% and 86.6% similarity with UAE-HKU30 strains and the product of this gene had 28 amino acids substituted in comparison to them. The NS7b gene of QdCoV/PL/G032/2015 had 97.1% and 92.3% homology on nucleotide and amino acid level, respectively. The nonstructural protein encoded by this gene differed from Middle Eastern viruses by nine amino acids (MILQHRHQS) inserted at the beginning and another ten substituted amino acids.

This study is the first description of the full-length genome sequence of QdCoV detected in the intestines of quails with enteric disease in Poland. Phylogenetic analyses showed that the Polish QdCoV/PL/G032/2015 branches separately but in the same genetic cluster as the Middle Eastern QdCoVs. The most variable part of the genome is the S gene, especially its fragment coding N-terminal domain of S protein responsible for receptor binding. The equivalent of this genome fragment (the S1 subunit) of the main representative of gammacoronaviruses, infectious bronchitis virus shows the highest diversity in the whole viral genome and is considered as critically important for the emergence of new virus genotypes, serotypes, and variants [3]. It seems that Polish QdCoV/PL/G032/2015 is another genotype/variant of QdCoV. Interestingly, unlike most of the genes, which have higher identities of amino acid than nucleotide sequences between the Polish and other quail coronavirus genomes, the levels of nt and aa identities of the S gene are similar. This observation might be the evidence for positive selection of aa changes within the S gene. Detection of QdCoV in quails with enteritis suggests that the virus might be responsible for observed disease symptoms but this requires additional in vivo studies. However, to do this, it would be necessary to isolate/propagate the virus on embryonated chicken or quail eggs which so far, despite many attempts, has been unsuccessful.

## Electronic supplementary material

Below is the link to the electronic supplementary material.

Supplementary material 1 (DOCX 16 KB)
