# Supplementary material for: Whole genome characterisation of quail deltacoronavirus detected in Poland
Source: Virus Genes. 2019 Feb 13;55(2):243–7. doi: 10.1007/s11262-019-01639-1 (PMC6458967; doi:10.1007/s11262-019-01639-1)
Supplement: Supplementary file 1 — Supplementary material 1 (DOCX 16 KB) [file 11262_2019_1639_MOESM1_ESM.docx]

**Supplemental Fig. 1.** Phylogenetic relationships between the complete genome of QdCoV/PL/G032/2015 strain from this study and other deltacoronaviruses. The tree was constructed using MEGA 7 using the neighbor-joining method and 1000 bootstrap replicates (bootstrap values shown on the tree). The scale bar indicates the number of nucleotide substitutions per site. GenBank accession numbers of the sequences are indicated in the parentheses. QdCoV/PL/G032/2015 strain determined in this study is marked with a black square.
